# Tongue pressure in sarcopenic and dynapenic elderly

**DOI:** 10.1590/2317-1782/e20240124en

**Published:** 2025-04-14

**Authors:** Ívina Thaiana de Almeida Menezes, Igor de Matos Pinheiro, Júlia Canto e Souza, Débora Matias dos Santos, Jaiele Freitas do Nascimento, Manuela Oliveira de Cerqueira Magalhães, Ana Caline Nóbrega

**Affiliations:** 1 Programa de Pós-graduação em Medicina e Saúde, Universidade Federal da Bahia – UFBA - Salvador (BA), Brasil.; 2 Obras Sociais Irmã Dulce – OSID - Salvador (BA), Brasil.; 3 Programa de Pós-graduação em Processos Interativos dos órgãos e Sistemas, Universidade Federal da Bahia – UFBA - Salvador (BA), Brasil.; 4 Departamento de Medicina Interna e Apoio Diagnóstico, Faculdade de Medicina da Bahia, Universidade Federal da Bahia – UFBA - Salvador (BA), Brasil.; 5 Departamento de Fonoaudiologia, Instituto Multidisciplinar em Saúde e Reabilitação, Universidade Federal da Bahia – UFBA - Salvador (BA), Brasil.

**Keywords:** Aged, Aging, Tongue, Pressure, Deglutition

## Abstract

**Purpose:**

We aimed to describe tongue pressure in sarcopenic and dynapenic older adults.

**Methods:**

An exploratory observational cross-sectional study was performed. Data were gathered from 29 institutionalized older adults (over 60 years old) and several methods were used in order to assess sarcopenia - handgrip strength (dynamometer), muscle mass (bioelectrical impedance analysis and calf circumference); physical performance (Short Physical Performance Balance); and tongue pressure (PLL Pró-Fono). For descriptive analysis, means and medians were described for quantitative variables and absolute and relative frequencies were described for qualitative variables. In the inferential analysis, Pearson's and Spearman's coefficients were used for correlation measurements and Chi-square and Fisher’s were used for association, 5% significance level.

**Results:**

Most patients were female (79.31%), with a median age of 81 years (IQR 12). Regarding diagnosis, 79.31% were sarcopenic, 17.24% were dynapenic and 3.45% did not present sarcopenia. Fifty-eight point six percent of patients presented low tongue pressure, being 88.2% aged 70-79 years old. Among sarcopenic older adults, 65.2% showed a decline in tongue pressure, while 40% showed similar results in the dynapenic group. There was a statistically significant positive correlation between handgrip and tongue pressure in the 70-79 years age group (p=0.03). With regards to women, there was an association between tongue pressure and sarcopenia (p=0.039) and a positive correlation between tongue pressure and handgrip (p=0.003).

**Conclusion:**

A decline in tongue strength was observed in the two studied groups, with worse outcomes in sarcopenic older adults.

## INTRODUCTION

Brazilian life expectancy has increased considerably, and the elderly have become increasingly representative^(1)^. Simultaneously with the aging process, institutionalization has become a reality, and these elderly people have worse adverse health effects when compared to those in the community^(2)^.

Some factors may be associated with functional incapacity in the elderly, including bodily changes such as loss of muscle strength (dynapenia) associated with a decline in muscle mass and function, a process known as sarcopenia. This condition considerably increases the likelihood of falls, fractures, physical disability and mortality^(3)^.

In the aging process, an association is found between sarcopenia, tongue strength and dysphagia^(4)^. Due to the reduction in generalized muscle mass and strength, the strength of the swallowing muscles is impaired and tongue pressure and sarcopenia may be directly related in the elderly, with poor outcomes and increased health costs^(5)^.

Considering that the institutionalized elderly are more fragile and vulnerable to comorbidities, the possibility of poor outcomes from sarcopenia and its relationship with swallowing alterations throughout aging, it is necessary to understand markers that can guide the speech therapy clinic for better intervention and functionality of the elderly. The aim of this study was therefore to describe tongue pressure in institutionalized elderly people with sarcopenia and dynapenia.

## METHODS

This is an observational, exploratory, cross-sectional study carried out with 29 elderly people institutionalized at the Júlia Magalhães Geriatrics and Gerontology Centre (CGGJM in Portuguese) of the Irmã Dulce Social Works, a philanthropic institution and reference center for the health care of the elderly in the city of Salvador, Bahia. The project was approved by the Research Ethics Committee, under Report No. 4.248.435, of the hospital where the study was carried out. Data collection began after the Informed Consent Form (ICF) had been read and signed.

The data was collected between July and December 2021 by previously trained researchers and employees of the long-term care facility for the elderly located in the Center.

The inclusion criteria applied were: elderly people of both sexes, aged 60 or over, residents of the CGGJM, who agreed to sign the ICF. Those whose data of interest to the study was incomplete or non-existent in the medical records, those diagnosed with or undergoing treatment for cancer, those who at the time of data collection had major infections (HIV, Zika virus, influenza, tuberculosis, pneumonia, meningitis, syphilis), burns or trauma and those who were unable to perform any stage of the tests, either because they did not understand the command or because they refused the procedure, were excluded. In addition, subjects with tracheostomies, whose nutrition was provided by enteral or parenteral means, were not included.

All participants were subjected to the following data collection procedures:

1. **Socio-demographic and clinical data**: age, gender (female or male), race/skin color, schooling (illiterate or based on the last grade studied by the elderly person: elementary, high school or college), history of drinking and smoking, clinical diagnosis and comorbidities and medications in use. The presence of two or more diseases was considered a comorbidity.
2. **Cognitive status:** assessed by the Mini-Mental State Examination (MMSE). For the diagnosis, the cut-off points were adjusted according to the level of education of the elderly person: ≤ 13 points - illiterate; ≤ 18 points - one to seven years of schooling; ≤ 26 points - more than eight years of schooling^(6)^.
3. **Diagnosis of dynapenia:** carried out using handgrip strength, using a Saehan® dynamometer, in accordance with the measurement recommendations of the American Society of Hand Therapists (ASHT). Three measurements were taken in the dominant hand, and the one with the highest value was recorded. Dynapenia was considered when the measurement was <27 kg for men and <16 kg for women. Elderly people with cognitive decline that made it impossible to understand the execution were considered dynapenia.
4. **Diagnosis of sarcopenia:** based on the revised European Consensus on Sarcopenia (2019), based on three components: low muscle strength (dynapenia), low muscle mass and low physical performance.
  1) *Low muscle strength (dynapenia)* was assessed by handgrip strength, using a Saehan dynamometer^(7)^, according to the ASHT measurement recommendations^(8)^. Three measurements were taken in the dominant hand and the one with the highest value was recorded. Muscle weakness was considered when the measurement was <27 kg for men and <16 kg for women^(9)^. For elderly people with cognitive decline that made it impossible to understand the execution, muscle weakness was considered.
  2) *Low muscle mass:* assessed using calf circumference (CC)^(3)^. CC was measured using a flexible, inelastic tape measure, with the participant sitting on a chair or hospital bed, forming a 90º angle with the knee and ankle. The measurement was taken laterally on the right side of the body, positioning the tape measure on the circumference of the calf, on the part with the largest protuberance. The reading was taken to the nearest millimeter and in duplicates, and the values obtained were averaged^(10)^. Low muscle mass was considered when the measurement was ≤ 33 cm for women and ≤ 34 cm for men^(11)^.
  3) *Low physical performance:* assessed using the Short Physical Performance Balance (SPPB). This is an exercise battery made up of three tests: 1) *Balance test* - the participant must be able to remain in each of the three positions for ten seconds: a) standing with feet together; b) standing with one foot partially forward; c) standing with one foot completely forward. For each, a score was given according to how long the participant managed to stay in the position; (2) *Chair test* - the participant was asked to get up and sit down from a chair five consecutive times, as quickly as possible. The score was given according to the time taken to complete the test; (3) *Four-meter speed test* - the participant had to walk, at a normal pace, a distance of four meters, demarcated by tapes fixed to the floor. The score was given according to the time taken to complete the test.

The final SPPB score was the total of the three tests and could vary from 0 to 12 points, with the following classification: 0 to 3 points: disability or poor ability; 4 to 6 points: low ability; 7 to 9 points: moderate ability; 10 to 12 points: good ability. The maximum score obtained in this battery of tests is 12 points and low performance was considered when ≤ 8 points were obtained^(3)^.

The findings of the sarcopenia assessment include the following outcomes: 1) Probable sarcopenia: reduced muscle strength; and 2) Sarcopenia (confirmed and severe): reduced strength together with reduced muscle mass, with or without reduced physical performance^(3)^.

1. **Assessment of muscle mass using Bioelectrical Impedance Analysis (BIA)**: in addition to CC, BIA was used as an additional method for assessing muscle mass, although it was not used to diagnose sarcopenia, since not all the elderly were able to perform the test. A Biodynamics model 310e was used. The measurements were taken using a tetrapolar cable arranged in pairs, with the electrodes attached to the hand and foot, on the right side of the body. The test was carried out with the subject lying on a non-conductive surface, in the supine position, with arms and legs abducted at 45°. The participants were instructed to follow the previous procedures, which included: fasting for four hours, not drinking alcohol 48 hours before the test, not doing intense exercise in the 12 hours before the test and emptying their bladder at least 30 minutes before the assessment. Based on the reactance (Xc) and resistance (R) data obtained by BIA, as well as weight, gender and age data, Appendicular skeletal muscle mass (ASM) was calculated using the formula by Sergi (2015): ASM (kg) = - 3.964 + (0.227 x RI) + (0.095 x weight) + (1.384 x gender) + (0.064 x Xc), where RI (resistive index) = height^2^/R, male gender = 1 and female gender = 2. Low muscle mass was considered when the result of the equation was <20 kg for men and <15 kg for women^(3)^.
2. **Tongue pressure measurement**: carried out by the speech therapist using the Pró-Fono portable BioFeedback device: lip and tongue pressure (PPL Pró-Fono, in Portuguese)^(12)^, registered with Anvisa 10368380047. The PLL Pró-Fono consists of a pressure sensor connected to an electronic board, both housed in a plastic case. The pressure sensor is connected to a flexible plastic tube connected to an air bulb device, which is connected to a flexible plastic tube connected to a pressure sensor, which detects the variations in air pressure transmitted and converts them into a Kilopascal (kPa) versus Time (s) graph^(12)^.

The measurements were started after calibrating the device connected to an Acer computer, model Aspire E5-571, year 2014. Calibration was carried out automatically by the instrument and the screen showed 0.0 kPa, meaning that the instrument had been successfully calibrated. The study participant was instructed to press the air bulb against the hard palate with maximum voluntary effort. Tongue pressure was measured three times and the maximum data recorded. The cut-off point for low tongue muscle strength was set at less than 20 kPa of pressure^(13)^.

At the end of the data collection, the data was entered into the SPSS for Windows software, version 4.1.0, and analyzed and possible associations and correlations were established. To characterize the sample, a descriptive analysis was carried out for the quantitative variables, using mean or median depending on the distribution of the data and standard deviation or interquartile range, depending on the measure of central tendency considered; in the case of the qualitative variables, their respective absolute and relative frequencies were presented. From an inferential point of view, Pearson's or Spearman's correlation tests were used to analyze associations between quantitative variables and, for associations between qualitative variables, the Chi-square or Fisher's test, considering the limitations of the data frequencies.

## RESULTS

The results obtained correspond to the analysis of data from 29 elderly people selected for the study (Table 1). All had ≥ two concomitant chronic diseases, a history of polypharmacy, malnutrition or were at nutritional risk and muscle weakness.

**Table 1:** Sociodemographic data and characterization of the sample in terms of chronic diseases, polypharmacy, nutritional status, muscle strength and sarcopenia

	No.	n (%)	Average	IQR
**Gender**				
Male	6	20.69		
Female	23	79.31		
**Age**				
			81.00	12.0
**Decade**				
60-69	2	6.90		
70-79	11	37.93		
80-89	12	41.38		
≥90	4	13.79		
**Race**				
White	10	34.48		
Brown	9	31.03		
Black	10	34.48		
**Cognition**				
Cognitive deficit	14	48.28		
No Cognitive Deficit	15	51.72		
**Education**				
Illiterate	6	20.69		
Elementary Incomplete	16	55.17		
Elementary School Complete	5	17.24		
High School Incomplete	-	-		
High School Complete				
	1	3.45		
College Incomplete	-	-		
College Complete	1	3.45		
**Chronic Diseases**				
Yes	29	100.00		
No	-	-		
**Polypharmacy** ^*^				
Yes	23	79.31		
No	6	20.69		
**Nutritional Condition**				
Malnutrition and risk	18	62.07		
Normal Nutrition Condition	11	37.93		
**CC Mass**				
Low Muscle Mass	24	82.76		
Normal Muscle Mass	5	17.24		
**Weak Hand Grip**				
Yes	28	96.55		
No	1	3.45		
**Strength of Tongue**				
Normal	12	41.38		
Weak	17	58.62	14.54	18.33
**Sarcopenia CC**				
No Sarcopenic	1	3.45		
Dynapenic	5	17.24		
Sarcopenic	23	79.31		

* Polypharmacy: continuous and concomitant use of three or more medications

Caption: IQR = Interquartile Range; CC = Calf Circumference

Source: Research Data

Among the 29 individuals selected, 23 were sarcopenic, of whom 95.6% had severe sarcopenia and 78.2% were female, 5 were dynapenic and only 1 did not have sarcopenia or dynapenia. Of the dynapenic group, 4 were female.

When it came to characterizing muscle mass, 82.7% had low muscle mass according to CC and 79.2% according to BIA. All the participants who showed impairment in the BIA also had the same outcome in the CC measurement. Furthermore, for both measurements, 100% of the elderly remained in the same sarcopenia classification group.

Of the elderly with low tongue pressure, 5.9% were under 70, 88.2% were aged between 70 and 89 and 5.9% were over 90, 82.3% were female.

The median tongue pressure value for participants aged between 60 and 69 was 54.21 kPa (IQR= 45.41), while the other decades had a value below 20kPa, which characterizes low tongue pressure.

Considering tongue pressure and dynamometry values, a reduction in handgrip strength was observed as tongue pressure values decreased. However, this positive correlation, observed by both CC and BIA, was not statistically significant.

**Figure 1:**
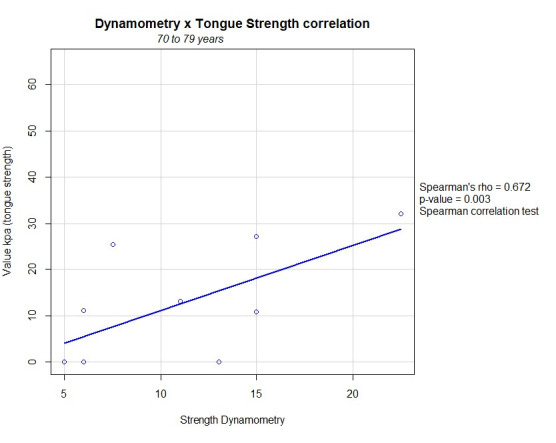
Linear correlation between tongue strength and generalized muscle strength in elderly individuals aged 70 to 79 years

Source: Research Data

In the elderly aged between 70 and 79, there was a positive correlation between tongue pressure and handgrip strength (p=0.03) (Figure 1).

When the sample was dichotomized by gender, 50% of the male group had low tongue strength and 60.8% of the female group had this condition.

**Figure 2:**
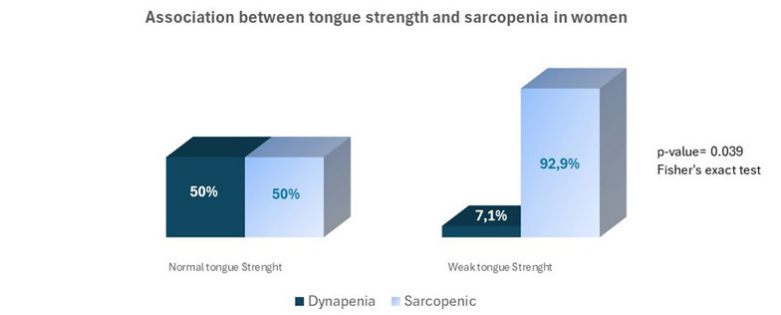
Association between tongue strength and sarcopenia in women

Source: Research Data

**Figure 3:**
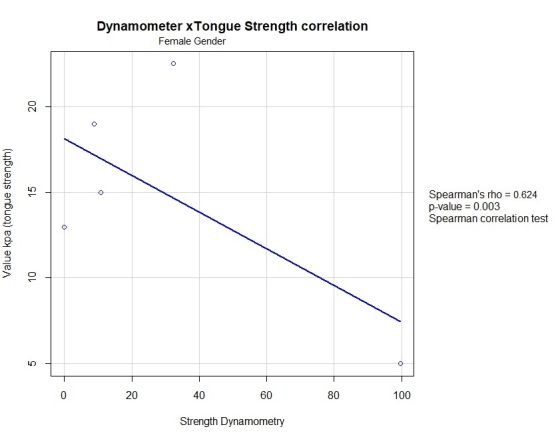
Linear correlation between handgrip strength and tongue strength in the female group

Source: Research Data

Of the 23 women, 18 were sarcopenic and 4 were dynapenic. In the first group, 72.2% had low tongue strength, reflecting an association between tongue strength and sarcopenia (p=0.039) (Figure 2) and a statistically significant correlation between tongue pressure and handgrip (p=0.003) (Figure 3).

Of the 6 male participants, 50% had low tongue pressure, of which 66.7% were sarcopenic and 33.3% were dynapenic. For this analysis, there was no association between tongue pressure and sarcopenia or increasing age.

Regarding the outcomes of sarcopenia and dynapenia, it was observed that in the dynapenic group, 60% of the participants had normal tongue pressure and 40% had reduced tongue pressure, while in the sarcopenic group it was observed that 65.2% of the elderly had a decline in tongue pressure and 34.8% had preserved tongue pressure.

## DISCUSSION

In this study, there was no statistically significant difference in tongue pressure between the sarcopenia and dynapenia groups. However, it was noted that in the sarcopenic group, tongue pressure is lower in women when compared to men and correlates with handgrip strength.

This lack of difference observed, even though the elderly sarcopenic group showed a higher frequency of low tongue pressure, may be due to the small number of dynapenic individuals in the group studied (n) and the older age of the sarcopenic group.

The frequency of low tongue pressure observed in the sarcopenic elderly group can be explained by the loss of muscle mass, which is not found in dynapenia, and which directly interferes with the proper functioning of the fibers, contributing to worse skeletal muscle strength parameters, one of which is the decline in tongue pressure^(14)^. Although the influence of muscle mass on tongue pressure has been little studied, it is known that there may be a positive correlation between these variables^(15)^.

Both sarcopenia and dynapenia can occur in the elderly population, but they don't necessarily occur linearly over time^(16)^. In the present study, more than half of the participants had low tongue pressure and muscle mass, which may be a potential risk factor for dysphagia^(17)^ and reflects the prevalence of impaired maximum isometric tongue pressure in the sarcopenic group.

It is known that there is an association between maximum tongue pressure and sarcopenia^(13)^. One possible reason is that the generalized decline in muscle mass and strength in sarcopenic elderly people also affects the muscles related to swallowing, such as the tongue^(18)^.

The mechanisms that lead to greater losses in muscle mass with ageing among different gender groups are unknown, but it has been suggested that sarcopenia is a greater public health problem for women, since they live longer and therefore have higher rates of disability. The “feminization” of population aging may be due to the greater demand for health services by this gender, while males are more exposed to risk factors for comorbidities and acute events^(19)^.

The Long-stay Institutions for the Elderly (ILPIs, in Portuguese) in this study had mostly female residents with sarcopenia. In addition, the women were older than the men, which may justify the association between these variables when comparing the two groups, since the decline in isometric tongue pressure can worsen with the ageing process^(20)^.

The gradual decline in tongue pressure in the elderly is associated with the process of presbyphagia and even individuals with no previous complaints may experience swallowing difficulties due to age-related changes^(21,22)^. However, although most swallowing disorders may be due to age-related changes, around 1/3 of this population may be accompanied by sarcopenia^(21)^.

Tongue pressure measurements in the elderly are commonly found in geriatric outpatient clinics^(23)^. However, this parameter has been discussed as a clinical marker of dysphagia and it is believed that reduced tongue muscle pressure is associated with unfavorable conditions in the institutionalized elderly, as well as subcomponents of sarcopenia^(18)^.

In addition to the change in muscle mass in sarcopenia, handgrip strength was also associated with a reduction in tongue pressure in the women's group. The decline in strength in the oropharyngeal structures may reflect the impairment of the overall skeletal musculature, disabilities and age-related difficulties, which reflects worse dynamometry parameters, correlated with the tongue pressure values in this study^(18,22)^. Thus, the association between tongue pressure and handgrip shows the need to include both measures in the clinical assessment of the elderly.

In addition, there are hypotheses that elderly people with cumulative deficiencies in multiple domains influence their swallowing functional reserve with increasing age^(24)^. Therefore, the correlation between reduced tongue pressure and a decline in handgrip may be explained by both sarcopenia and low functional reserve in older people^(25)^.

Thus, the results found in the group of elderly women may be related to longevity and the number of participants of this gender. The fact that there were fewer men may have interfered with the results. There is a scarcity of studies comparing gender between sarcopenic elderly people and tongue strength. The publications that reflect this difference do not present sarcopenia as a diagnosis^(26)^.

As for the correlation between handgrip strength and tongue pressure found in this study, in the group of individuals aged between 70 and 79 it was observed that the elderly in this age group were also mostly women and had been diagnosed with sarcopenia.

Although it cannot be confirmed that sarcopenia precedes the decline in tongue and handgrip pressure, this study shows a correlation between these variables in women. Further studies are needed into the impact of this impairment on swallowing function in sarcopenic and dynapenic elderly people.

The change in tongue pressure in the dynapenic group was not significant, suggesting that, in this study, the loss of muscle mass had worse outcomes than the loss of strength alone in the elderly. On the other hand, some publications show that dynapenia, associated with the aging process, has a functional impact on the elderly and that the reduction in tongue pressure in this population may be related to the functional decline of swallowing, interfering with both muscle movement and pressure exerted^(27,28)^.

Our results suggest that sarcopenia leads to unfavorable outcomes in the elderly, both in terms of overall skeletal muscle mass and generalized strength, as well as oral aspects, which corroborates other studies that have reaffirmed the influence of loss of generalized muscle mass and strength, as well as handgrip strength in the investigation of reduced tongue pressure and oral sarcopenia^(18,29)^.

In relation to the different methods used in this study to assess muscle mass, there was no difference in the classification of sarcopenia, showing that both CC and BIA measurements had the same outcomes.

The fact that there was no difference in the characterization of muscle mass and the diagnosis of sarcopenia using CC and BIA shows that the first method can be used more routinely by professionals, as it is easy to train and more accessible in clinical practice.

CC is used as a clinical marker to measure muscle mass in the screening and diagnosis of sarcopenia. However, this measurement is dependent on other variables such as muscle strength and age^(30,31)^, which were also analyzed in this study.

### Study limitations

This study described tongue pressure in sarcopenic and dynapenic individuals based on a sample of elderly residents of a long-term care facility. It is therefore not possible to generalize the results to the entire elderly population.

The population analyzed in the sarcopenic elderly group was older than the dynapenic group, which contributes to worse outcomes in terms of tongue pressure impairment. Furthermore, an important limitation refers to the sample size of the dynapenic group, which was smaller than that of the sarcopenic elderly group.

The sample size can be considered a limiting factor, which suggests that these outcomes are limited to the population in question.

## CONCLUSION

A decline in tongue pressure was identified in this sample in both the sarcopenia and dynapenia groups, but with a worse outcome in the sarcopenic patients. In addition, tongue and handgrip strength in the women's group and in the elderly aged between 70 and 79 were moderately correlated, suggesting that generalized muscle weakness may increase the risk of tongue pressure impairment in older women.

The limitations of this study compromise the generalizability of the results, and further research is needed to confirm these preliminary conclusions.
