# Supplementary material for: Tongue pressure in sarcopenic and dynapenic elderly
Source: Codas. 2025 Apr 14;37(3):e20240124. doi: 10.1590/2317-1782/e20240124en (PMC11999703; doi:10.1590/2317-1782/e20240124en)
Supplement: Translation

# Translation: Pressão de língua em idosos sarcopênicos e dinapênicos

**DOI:** 10.1590/2317-1782/e20240124pt

**Authors:** Ívina Thaiana de Almeida Menezes, Igor de Matos Pinheiro, Júlia Canto e Souza, Débora Matias dos Santos, Jaiele Freitas do Nascimento, Manuela Oliveira de Cerqueira Magalhães, Ana Caline Nóbrega

**Affiliations:** 1 Programa de Pós-graduação em Medicina e Saúde, Universidade Federal da Bahia – UFBA - Salvador (BA), Brasil.; 2 Obras Sociais Irmã Dulce – OSID - Salvador (BA), Brasil.; 3 Programa de Pós-graduação em Processos Interativos dos órgãos e Sistemas, Universidade Federal da Bahia – UFBA - Salvador (BA), Brasil.; 4 Departamento de Medicina Interna e Apoio Diagnóstico, Faculdade de Medicina da Bahia, Universidade Federal da Bahia – UFBA - Salvador (BA), Brasil.; 5 Departamento de Fonoaudiologia, Instituto Multidisciplinar em Saúde e Reabilitação, Universidade Federal da Bahia – UFBA - Salvador (BA), Brasil.

**Keywords:** Idoso, Envelhecimento, Língua, Pressão, Deglutição

## Abstract

**Objetivo:**

Descrever pressão de língua em idosos com sarcopenia e dinapenia.

**Método:**

Estudo observacional, exploratório, de delineamento transversal. Coletaram-se dados de 29 idosos institucionalizados (>60 anos) e realizou-se avaliação de sarcopenia – força de preensão manual (dinamômetro), massa muscular (bioimpedância elétrica e circunferência de panturrilha) e de desempenho físico - e de pressão de língua. Na análise descritiva, utilizaram-se as médias e medianas para as variáveis quantitativas e frequências absolutas e relativas para as qualitativas. Na análise inferencial, utilizou-se Pearson ou Spearman para os testes de correlação e Qui-quadrado ou Fisher para os de associação, nível de significância de 5%.

**Resultados:**

A amostra se caracterizou por ser predominantemente feminina (79,31%), com idade mediana de 81 anos (QI=12). Identificou-se que 79,31% eram sarcopênicos, 17,24% dinapênicos e 3,45% sem sarcopenia. De toda amostra, 58,6% apresentaram baixa pressão de língua, sendo 88,2% com idade entre 70 e 79 anos. No grupo de idosos sarcopênicos, 65,2% apresentaram declínio na pressão de língua e no grupo dos dinapênicos, 40%. Observou-se correlação positiva e estatisticamente significante entre força de preensão manual e pressão de língua nos idosos entre 70 e 79 anos (p=0,03). No grupo das mulheres, ocorreu associação entre pressão de língua e sarcopenia (p=0,039) e correlação positiva entre pressão de língua e preensão manual (p=0,003).

**Conclusão:**

O declínio da pressão de língua foi observado nos dois grupos estudados, sendo identificado pior desfecho no grupo com sarcopenia.

## INTRODUÇÃO

A expectativa de vida brasileira vem aumentando consideravelmente e os idosos têm ganhado uma representatividade cada vez maior^(1)^. Simultaneamente ao processo de envelhecimento, a institucionalização tem sido uma realidade e esses idosos apresentam piores efeitos adversos de saúde quando comparados aos da comunidade^(2)^.

Alguns fatores podem estar associados a incapacidade funcional em idosos, dentre eles as mudanças corporais como perda de força muscular (dinapenia) associado ao declínio de massa e função muscular, processo chamado de sarcopenia. Essa é uma condição que aumenta consideravelmente a probabilidade de risco de quedas, fraturas, incapacidade física e mortalidade^. (3)^

No processo do envelhecimento, é encontrada uma associação entre sarcopenia, força de língua e disfagia^(4)^. Em detrimento da redução de massa e força muscular generalizada, a força dos músculos da deglutição é prejudicada e a pressão de língua e sarcopenia podem apresentar uma relação direta nos idosos, com desfechos ruins e aumento nos custos de saúde^(5)^.

Considerando os idosos institucionalizados em condição de maior fragilidade e vulnerabilidade para comorbidades, possibilidade de desfechos ruins da sarcopenia e sua relação com as alterações da deglutição ao longo do envelhecimento, faz-se necessário compreender marcadores que possam nortear a clínica fonoaudiológica para melhor intervenção e funcionalidade do idoso. Portanto, o objetivo deste estudo é descrever a pressão de língua em idosos institucionalizados, com sarcopenia e dinapenia.

## MÉTODO

Trata-se de um estudo observacional, exploratório, de delineamento transversal, realizado com 29 idosos institucionalizados no Centro de Geriatria e Gerontologia Júlia Magalhães (CGGJM) das Obras Sociais Irmã Dulce, instituição filantrópica e centro de referência na atenção à saúde da pessoa idosa da cidade de Salvador, Bahia. O projeto foi aprovado pelo Comitê de Ética em Pesquisa, com o Parecer n. 4.248.435, do Hospital onde o estudo foi desenvolvido. A coleta de dados teve início após a leitura e assinatura do Termo de Consentimento Livre e Esclarecido (TCLE).

Os dados foram coletados entre julho e dezembro de 2021 por pesquisadores previamente treinados e colaboradores da instituição de longa permanência para idosos, locada no referido Centro.

Utilizou-se como critérios de inclusão: idosos de ambos os sexos, com idade igual ou maior a 60 anos, residentes do CGGJM, e que concordaram em assinar o Termo de Consentimento Livre e Esclarecido. Foram excluídos aqueles cujos dados de interesse do estudo estavam incompletos ou inexistentes nos prontuários, os diagnosticados ou em tratamento de câncer, os que no momento da coleta de dados apresentavam grandes infecções (HIV, Zika vírus, influenza, tuberculose, pneumonia, meningite, sífilis), queimaduras ou traumas e, ainda aqueles pela impossibilidade de execução de alguma etapa dos testes, seja por falta de compreensão do comando ou por recusa do procedimento. Além disso, não foram incluídos sujeitos com traqueostomia, cuja nutrição era fornecida por via enteral ou parenteral.

Todos os participantes foram submetidos aos seguintes procedimentos para levantamento de dados:

1. **Dados sociodemográficos e clínicos:** idade, sexo (feminino ou masculino), raça/cor da pele, escolaridade (analfabeto ou com base na última série estudada pelo idoso em: ensino básico, médio ou superior), histórico de etilismo e tabagismo, diagnóstico clínico e comorbidades e medicações em uso. A presença de duas ou mais doenças foi considerada comorbidade.
2. **Estado cognitivo –** avaliado pelo Mini-Exame do Estado Mental (MEEM). Para o diagnóstico, os pontos de corte foram ajustados de acordo com o nível de escolaridade do idoso: ≤ 13 pontos – analfabeto; ≤ 18 pontos – um a sete anos de escolaridade; ≤ 26 pontos – mais de oito anos de escolaridade^(6)^.
3. **Diagnóstico de dinapenia** - realizado por meio da força de preensão manual, utilizando-se o dinamômetro da marca Saehan®, conforme as recomendações de mensuração da American Society of Hand Therapists (ASHT). Realizaram-se três mensurações na mão dominante, sendo registrada a de maior valor. Considerou-se dinapenia quando a medida foi <27 kg para homens e <16 kg para mulheres. Para os idosos com declínio cognitivo que impossibilitasse a compreensão da execução, foi considerada dinapenia.
4. **Diagnóstico de sarcopenia** – baseado no Consenso Europeu de Sarcopenia revisado (2019), a partir de três componentes: baixa força muscular (dinapenia), baixa massa muscular e baixo desempenho físico.
  1) *Baixa força muscular (dinapenia*) foi avaliada pela força de preensão manual, utilizando-se o dinamômetro da marca Saehan^(7)^, conforme as recomendações de mensuração da ASHT^(8)^. Foram realizadas três mensurações na mão dominante e registrada a de maior valor. Foi considerada fraqueza muscular quando a medida foi <27 kg para homens e <16 kg para mulheres^(9)^. Para os idosos com declínio cognitivo que impossibilitasse a compreensão da execução, foi considerada fraqueza muscular.
  2) *Baixa massa muscular*: avaliada por meio da circunferência da panturrilha (CP).^(3)^A CP: foi aferida com fita métrica flexível e inelástica, com o participante sentado em uma cadeira ou na cama hospitalar, formando um ângulo de 90º com o joelho e o tornozelo. A medida foi realizada lateralmente, do lado direito do corpo, posicionando-se a fita métrica na circunferência da panturrilha, na parte com maior protuberância. A leitura foi realizada no milímetro mais próximo e em duplicatas, realizando-se a média dos valores obtidos^(10)^. Considerou-se baixa massa muscular quando a medida foi ≤ 33 cm para as mulheres e ≤ 34 cm para os homens^(11)^.
  3) *Baixo desempenho físico*: foi avaliada por meio da *Short Physical Performance Balance* (SPPB). Trata-se de uma bateria de exercícios composta por três testes: 1) *Teste de equilíbrio* - o participante deve conseguir manter-se em cada uma das três posições por dez segundos: a) em pé com os pés juntos; b) em pé com um pé parcialmente à frente; c) em pé com um pé totalmente à frente. Para cada uma, foi dada uma pontuação de acordo com o tempo que o participante conseguiu manter-se na posição. (2) *Teste da cadeira* - foi solicitado ao participante que levantasse e sentasse de uma cadeira cinco vezes consecutivas, o mais rápido que conseguir. A pontuação foi dada de acordo com o tempo de realização do teste. (3) *Teste de velocidade de quatro metros* - o participante deveria caminhar, em passo habitual, uma distância de quatro metros, demarcados por fitas fixas ao chão. A pontuação foi dada de acordo com o tempo de realização do teste.

A pontuação final do SPPB foi dada pela soma dos três testes e poderia variar de 0 a 12 pontos, sendo atribuída a seguinte classificação: zero a 3 pontos: incapacidade ou capacidade ruim; 4 a 6 pontos: baixa capacidade; 7 a 9 pontos: capacidade moderada; 10 a 12 pontos: boa capacidade. A pontuação máxima obtida nessa bateria de testes é de 12 pontos e foi considerada baixa performance quando obtinham-se ≤ 8 pontos^(3)^.

Para os achados da avaliação de sarcopenia, encontram-se os seguintes desfechos: 1) Sarcopenia provável: redução da força muscular; e 2) Sarcopenia (confirmada e severa): redução da força em conjunto com redução da massa muscular, com ou sem redução desempenho físico^(3)^.

1. **Avaliação da massa muscular por meio da bioimpedância elétrica (BIA)** – além da CP, a BIA foi utilizada como método adicional para avaliação da massa muscular, embora não tenha sido utilizada para o diagnóstico da sarcopenia, uma vez que nem todos os idosos foram capazes de realizar o exame. Utilizou-se o aparelho da marca *Biodynamics*, modelo 310e. As medidas foram realizadas por meio de cabo tetrapolar disposto aos pares, com os eletrodos fixados na mão e no pé, do lado direito do corpo. O teste foi realizado com o indivíduo deitado sobre uma superfície não-condutora, na posição supina, com braços e pernas abduzidos a 45°. Os participantes foram orientados a seguir os procedimentos prévios, ou seja: jejum absoluto de quatro horas, não ingerir álcool 48h antes do teste, não realizar exercício intenso nas 12 horas que antecedem o teste e esvaziar a bexiga pelo menos 30 minutos antes da avaliação. A partir dos dados de reactância (Xc) e resistência (R) obtidos pela BIA, bem como dados de peso, sexo e idade, foi calculada a massa muscular apendicular (MMA), a partir da fórmula de Sergi (2015): MMA (kg) = - 3.964 + (0.227 x RI) + (0.095 x peso) + (1.384 x sexo) + (0.064 x Xc), sendo RI (índice resistivo) = altura^2^/R, sexo masculino = 1 e sexo feminino = 2. Foi considerada baixa massa muscular quando o resultado da equação foi <20 kg para homens e <15 kg para mulheres^(3)^.
2. **Mensuração de pressão de língua** – realizada pela fonoaudióloga com o aparelho portátil de BioFeedback da marca Pró-Fono: pressão de lábios e de língua (PPL Pró-Fono)^(12)^, registro da Anvisa 10368380047. O PLL Pró-Fono é formado por um sensor de pressão conectado a uma placa eletrônica, sendo ambos acondicionados em um gabinete plástico. O sensor de pressão é ligado a um tubo plástico flexível conectado a um dispositivo de bulbo de ar, o qual está conectado a um tubo plástico flexível, ligado a um sensor de pressão, que detecta as variações da pressão do ar transmitidas e as converte em um Gráfico Kilopascal (kPa) *versus* Tempo (s)^(12)^.

As medições foram iniciadas após calibração do aparelho conectado a um computador da marca Acer, modelo Aspire E5-571, ano 2014. A calibração foi realizada automaticamente pelo instrumento e a tela mostrou 0,0 kPa, o que significa que o instrumento fora calibrado com sucesso. O participante do estudo foi orientado a realizar uma pressão no bulbo de ar contra o palato duro, com esforço voluntário máximo. A pressão da língua foi medida três vezes e os dados máximos foram registrados. Estabeleceu-se o ponto de corte para baixa força muscular de língua num valor inferior a 20 kPa de pressão^(13)^.

No final da coleta, os dados lançados no *software* SPSS para Windows, versão 4.1.0, foram analisados e, posteriormente, estabelecidas possíveis associações e correlações. Para caracterização da amostra, realizou-se análise descritiva para as variáveis quantitativas, utilizando média ou mediana dependendo da distribuição dos dados e desvio-padrão ou intervalo interquartil, dependendo da medida de tendência central considerada; no caso das variáveis qualitativas, foram apresentadas suas respectivas frequências absolutas e relativas. Do ponto de vista inferencial, utilizaram-se os testes de correlação de Pearson ou Spearman para análise das associações entre as variáveis quantitativas e para associação entre as variáveis qualitativas, o teste Qui-quadrado ou Fisher considerando as limitações das frequências dos dados.

## RESULTADOS

Os resultados obtidos correspondem à análise de dados de 29 idosos selecionados para estudo (Tabela 1). Todos apresentavam ≥ duas doenças crônicas concomitantes, histórico de polifarmácia, desnutrição ou estavam em risco nutricional e fraqueza muscular.

**Tabela 1:** Dados sociodemográficos e caracterização da amostra quanto doenças crônicas, polifarmácia, estado nutricional, força muscular e sarcopenia

	n	n (%)	Mediana	IQ
**Sexo**				
Masculino	6	20.69		
Feminino	23	79.31		
**Idade**				
			81.00	12.0
**Décadas**				
60-69	2	6.90		
70-79	11	37.93		
80-89	12	41.38		
≥90	4	13.79		
**Raça**				
Branca	10	34.48		
Parda	9	31.03		
Negra	10	34.48		
**Cognição**				
Déficit cognitivo	14	48.28		
Sem déficit cognitivo	15	51.72		
**Escolaridade**				
Analfabeto	6	20.69		
Fundamental incompleto	16	55.17		
Fundamental completo	5	17.24		
Ensino médio incompleto	-	-		
Ensino médio completo	1	3.45		
Superior incompleto	-	-		
Superior completo	1	3.45		
**Doenças crônicas**				
Sim	29	100.00		
Não	-	-		
**Polifarmácia** *				
Sim	23	79.31		
Não	6	20.69		
**Estado nutricional**				
Desnutrição e risco	18	62.07		
Estado nutricional normal	11	37.93		
**Massa CP**				
Baixa massa muscular	24	82.76		
Massa muscular normal	5	17.24		
**Fraqueza de preensão palmar**				
Sim	28	96.55		
Não	1	3.45		
**Força de língua**				
Normal	12	41.38		
Fraca	17	58.62	14.54	18.33
**Sarcopenia CP**				
Não sarcopênico	1	3.45		
Dinapênicos	5	17.24		
Sarcopênicos	23	79.31		

* Polifarmácia: uso contínuo e concomitante de três ou mais medicamentos

Legenda: IQ = Intervalo Interquartil; CP = Circunferência da panturrilha

Fonte: Dados da pesquisa

Entre os 29 indivíduos selecionados, 23 eram sarcopênicos, dos quais 95,6% tinham sarcopenia severa e 78,2% eram do sexo feminino, 5 eram dinapênicos e apenas 1 não apresentava sarcopenia ou dinapenia. No grupo dos dinapênicos,4 eram do sexo feminino.

Para caracterização da massa muscular, observou-se que 82,7% apresentaram baixa massa muscular pela medida de CP e 79,2% pela BIA. Todos os participantes que apresentaram comprometimento na BIA também obtiveram o mesmo desfecho na mensuração da CP. Além disso, para as duas medidas, 100% dos idosos se mantiveram no mesmo grupo de classificação de sarcopenia.

Dos idosos que apresentam pressão de língua baixa, 5,9% tinham menos de 70 anos, 88,2% estavam na faixa etária entre 70 e 89 anos e 5,9% acima de 90 anos, sendo 82,3% do sexo feminino.

O valor mediano da pressão de língua dos participantes entre 60 e 69 anos foi de 54,21 kPa (QI= 45,41), enquanto as demais décadas apresentaram um valor abaixo de 20kPa, o qual caracteriza baixa pressão de língua.

Considerando os valores de pressão de língua e da dinamometria, observou-se uma redução na força de preensão manual à medida que os valores da pressão de língua decresciam. No entanto, essa correlação positiva, observada tanto pela CP quanto pela BIA, não apresentou significância estatística.

Nos idosos com idade entre 70 e 79 anos, observou-se correlação positiva entre valor de pressão de língua e força de preensão manual (p=0,03) (Figura 1).

**Figura 1:** Correlação linear entre força de língua e força muscular generalizada em idosos de 70 a 79 anos

Fonte: Dados da pesquisa

Quando dicotomizada a amostra em gênero, no grupo masculino 50% apresentam baixa força de língua e no feminino 60,8% expressavam esta condição.

Das 23 mulheres, 18 eram sarcopênicas e 4 eram dinapênicas. No primeiro grupo foi observado que 72,2% apresentavam força de língua reduzida, refletindo uma associação entre força de língua e sarcopenia (p=0,039) (Figura 2) e uma correlação entre pressão de língua e preensão manual estatisticamente significante (p=0,003) (Figura 3***).***

**Figura 2:** Associação entre força de língua e sarcopenia no grupo feminino

**Fonte:** Dados da pesquisa

**Figura 3:** Correlação linear entre força de preensão manual e força de língua no grupo feminino

Fonte: Dados da pesquisa

Dos 6 participantes homens, 50% apresentavam baixa pressão de língua, dos quais eram 66,7% sarcopênicos e 33,3% dinapênicos. Para esta análise, não foi notada associação entre pressão de língua e sarcopenia ou aumento da idade.

Em relação aos desfechos de sarcopenia e dinapenia, observou-se que, no grupo dos dinapênicos, 60% participantes apresentaram pressão normal de língua e 40% reduzida, enquanto que no grupo dos sarcopênicos observou-se que 65,2% dos idosos apresentavam declínio na pressão de língua e 34,8% tinham pressão de língua preservada.

## DISCUSSÃO

Neste estudo, não foi observada diferença estatisticamente significante de pressão de língua entre os grupos de sarcopenia e dinapenia. No entanto, notou-se que no grupo dos sarcopênicos, a pressão de língua é mais baixa nas mulheres quando comparada a dos homens e se correlaciona com a força de preensão manual.

Essa falta de diferença observada, mesmo com grupo de idosos de sarcopênicos apresentando uma maior frequência de baixa pressão de língua, pode ser consequência do reduzido número dinapênicos no grupo estudado (n) e da idade mais avançada no grupo dos sarcopênicos.

A frequência da baixa pressão de língua observada no grupo dos idosos sarcopênicos pode ser justificada pela perda de massa muscular, a qual não é encontrada na dinapenia e que interfere diretamente no funcionamento adequado das fibras, contribuindo para piores parâmetros de força muscular esquelética, sendo um deles o declínio na pressão de língua^(14)^. Embora, a influência da massa muscular na pressão de língua seja pouco estudada, sabe-se que pode existir uma correlação positiva entre essas variáveis^(15)^.

Tanto a sarcopenia, quanto a dinapenia, podem ocorrer na população idosa, no entanto não acontecem necessariamente de forma linear ao longo do tempo^(16)^. No presente estudo, mais da metade dos participantes apresentaram baixa pressão de língua e de massa muscular, o que pode ser um fator de risco potencial para disfagia^(17)^ e reflita a prevalência de comprometimento da pressão máxima isométrica de língua no grupo dos sarcopênicos.

Sabe-se que existe uma associação entre pressão máxima de língua e sarcopenia^(13)^. Uma possível razão é que o declínio generalizado de massa e força muscular nos idosos sarcopênicos também afeta os músculos relacionados à deglutição, como por exemplo a língua^(18)^.

Os mecanismos que levam a maiores perdas de massa muscular com o envelhecimento entre os grupos de gêneros distintos são desconhecidos, porém tem sido sugerido que a sarcopenia é um problema de saúde pública maior para as mulheres, uma vez que elas vivem mais e por isso têm maiores taxas de incapacidade. A “feminilização” do envelhecimento populacional pode ser justificado devido à maior procura dos serviços de saúde por esse gênero, enquanto que o sexo masculino tem maior exposição a fatores de risco para as comorbidades e eventos agudos^(19)^.

A ILPI deste estudo apresentava, na sua maioria, residentes do sexo feminino e com sarcopenia. Além disso, as mulheres tinham idade mais avançada que os homens, o que pode justificar a associação entre essas variáveis, quando comparados os dois grupos, uma vez que o declínio da pressão isométrica de língua pode piorar com o processo do envelhecimento^(20)^.

O declínio gradual da pressão de língua nos idosos está associado ao processo de presbifagia e mesmo em indivíduos sem queixas prévias podem apresentar dificuldades na deglutição devido a modificações relacionadas ao envelhecimento^(21,22)^. No entanto, embora a maioria dos distúrbios da deglutição possa ser devido a alterações relacionadas à idade, cerca de 1/3 dessa população pode ser acompanhado da sarcopenia^(21)^.

A avaliação da mensuração da pressão de língua em idosos é encontrada comumente em ambulatórios de geriatria^(23)^. No entanto, tem-se discutido este parâmetro como um marcador clínico da disfagia e acredita-se que a redução da pressão muscular da língua esteja associada a condições desfavoráveis nos idosos institucionalizados, bem como a subcomponentes da sarcopenia^(18)^.

Além da alteração de massa muscular na sarcopenia, a força de preensão manual também apresentou associação com a redução na pressão de língua no grupo das mulheres. O declínio de força nas estruturas orofaríngeas pode ser reflexo do comprometimento da musculatura esquelética global, das incapacidades e dificuldades relacionadas à idade, o que reflete piores parâmetros na dinamometria, correlacionados aos valores de pressão de língua do presente estudo^(18,22)^. Assim, a associação entre pressão de língua e de preensão manual mostra a necessidade de incluir ambas medidas na avaliação clínica do idoso.

Além disso, há hipóteses de que idosos com deficiências cumulativas em múltiplos domínios têm afetada a reserva funcional da deglutição com o aumento da idade^(24)^ Sendo assim, a correlação entre redução da pressão de língua e declínio da preensão manual pode ser justificada tanto pela sarcopenia quanto pela baixa reserva funcional em idosos mais longevos^(25)^.

Dessa forma, os resultados encontrados no grupo das idosas podem estar relacionados com a longevidade e o número de participantes desse gênero. Já nos homens pode ter interferido nos resultados encontrados o fato de serem em menor número. Há escassez de estudos quanto à comparação de gênero entre idosos sarcopênicos e força de língua. As publicações que refletem essa diferença não apresentam a sarcopenia como diagnóstico^(26)^.

Quanto à correlação da força de preensão manual e pressão de língua encontrada neste estudo, no grupo de indivíduos entre 70 e 79 anos observou-se que os idosos dessa faixa etária eram também na sua maioria mulheres e apresentavam o diagnóstico de sarcopenia.

Embora não se possa ratificar que a sarcopenia precede o declínio da pressão de língua e de preensão manual, este estudo demonstra uma correlação entre essas variáveis no sexo feminino. Estudos adicionais são necessários para o impacto desse comprometimento na função de deglutição em idosos sarcopênicos e dinapênicos.

A alteração de pressão de língua no grupo dos dinapênicos não foi significante, fato sugestivo que, neste estudo, a perda de massa muscular apresentou piores desfechos do que a perda de força isolada no idoso. Em contrapartida, algumas publicações mostram que a dinapenia, associada ao processo do envelhecimento, apresenta impacto funcional nos idosos e que a redução da pressão de língua nessa população pode estar relacionada com o declínio funcional da deglutição, interferindo tanto na movimentação dos músculos, quanto na pressão exercida^(27,28)^.

Os nossos resultados sugerem que a sarcopenia gera desfechos desfavoráveis nos idosos tanto em relação a massa muscular esquelética global e força generalizada quanto aos aspectos orais, o que corrobora com outros estudos que reafirmam influência da perda de massa e força muscular generalizada, bem como a preensão de força manual na investigação da redução da pressão de língua e sarcopenia oral^(18,29)^.

Em relação aos diferentes métodos de investigação deste estudo, quanto às medidas de avaliação de massa muscular, não se observou diferença de classificação de sarcopenia, retratando que tanto a mensuração pela CP, quanto pela BIA apresentaram os mesmos desfechos.

A não diferença observada na caracterização da massa muscular e no diagnóstico de sarcopenia pela utilização do CP e da BIA mostra que o primeiro método pode ser utilizado de forma mais rotineira pelos profissionais, por ser de fácil treinamento e mais acessível na prática clínica

A CP é utilizada como marcador clínico para mensuração de massa muscular no rastreio e no diagnóstico da sarcopenia. No entanto, esta medida é dependente de outras variáveis como por exemplo força muscular e idade^(30,31)^, também analisadas no presente estudo.

### Limitação do estudo

O presente estudo descreveu a pressão de língua em indivíduos sarcopênicos e dinapênicos a partir de uma amostra de idosos residentes de uma instituição de longa permanência. Dessa forma, não é possível generalizar o resultado para toda população idosa.

A população analisada no grupo dos idosos sarcopênicos tinha idade mais avançada que no grupo dos dinapênicos, o que contribui para piores desfechos em relação ao comprometimento da pressão de língua. Além disso, uma limitação importante se refere ao tamanho da amostra do grupo dos dinapênicos, a qual foi menor em relação ao grupo dos idosos sarcopênicos.

O tamanho da amostra pode ser considerado um fator limitante, o que se sugere que esses desfechos se resumem apenas para a população em questão.

## CONCLUSÃO

O declínio da pressão de língua foi identificado nesta amostra tanto no grupo com sarcopenia, quanto com dinapenia, porém com pior desfecho nos pacientes sarcopênicos. Além disso, a força de língua e de preensão manual no grupo das mulheres e nos idosos entre 70 e 79 anos correlacionaram-se moderadamente, o que sugere que a fraqueza muscular generalizada pode aumentar o risco de comprometimento da pressão de língua em mulheres mais longevas.

As limitações deste estudo comprometem a generalização dos resultados, sendo necessárias mais investigações para confirmar estas conclusões preliminares.
